# Differential Predictors and Clinical Implications Associated With Long-Term Survivors in IDH Wildtype and Mutant Glioblastoma

**DOI:** 10.3389/fonc.2021.632663

**Published:** 2021-05-13

**Authors:** Haihui Jiang, Kefu Yu, Yong Cui, Xiaohui Ren, Mingxiao Li, Guobin Zhang, Chuanwei Yang, Xuzhe Zhao, Qinghui Zhu, Song Lin

**Affiliations:** ^1^ Department of Neurosurgery, Beijing Tiantan Hospital, Capital Medical University, Beijing, China; ^2^ National Clinical Research Center for Neurological Diseases, Center of Brain Tumor, Beijing Institute for Brain Disorders and Beijing Key Laboratory of Brain Tumor, Beijing, China; ^3^ Department of Pharmacy, Beijing Tiantan Hospital, Capital Medical University, Beijing, China; ^4^ Beijing Neurosurgical Institute, Capital Medical University, Beijing, China

**Keywords:** glioblastoma, long-term survivor, IDH, precision medicine, treatment

## Abstract

**Background:**

Glioblastoma (GBM) is the most aggressive intracranial tumor which can be divided into two subtypes based on status of isocitrate dehydrogenase (IDH). A small fraction of patients after receiving standard treatment can be long-term survivors (LTS). This study was designed to disclose the predictors and clinical implications associated with LTS in IDH wildtype and mutant GBM.

**Methods:**

Patients who survived beyond five years after diagnosis of GBM were defined as LTS, while those with a survival less than one year were defined as short-term survivors (STS). A total of 211 patients with diagnosis of GBM in Beijing Tiantan Hospital from January 2007 to January 2015 were enrolled, including 44 (20.9%) LTS and 167 (79.1%) STS. The clinical, radiological and molecular features between groups were systematically compared.

**Results:**

Compared with STS, LTS were a subgroup of patients with a younger age at diagnosis (*P*=0.006), a higher KPS score (*P*=0.011), higher rates of cystic change (*P*=0.037), O^6^-methylguanine-DNA methyltransferase (MGMT) promoter methylation (*P*=0.007), and IDH mutation (*P*=0.049), and more likely to have undergone gross total resection (*P*<0.001). Survival analysis demonstrated that LTS with wildtype IDH conferred a longer progression-free survival (66.0 *vs.* 27.0 months, *P*=0.04), but a shorter post-progression survival (46.5 months *vs.* not reached, *P*=0.0001) than those of LTS with mutant IDH. LTS with mutant IDH showed a trend towards increased survival after receiving re-operation (*P*=0.155) and reirradiation (*P*=0.127), while this clinical benefit disappeared in the subset of LTS with wildtype IDH (*P*>0.05).

**Conclusion:**

The prognostic value and therapeutic implications associated with LTS in GBM population significantly differed on the basis of IDH status. Our findings provide a new approach for physicians to better understand the two subtypes of GBM, which may assist in making more tailored treatment decisions for patients.

## Introduction

Glioblastoma (GBM) is one of the worldwide intractable malignancies in adults (1). Despite advances in the therapeutic regimens during past few decades, the clinical outcomes of patients with GBM have not been substantially improved (2–4). It is reported that most of patients will progress within one year after resection, and the median survival is less than two years (4). Although the survival rate of GBM remains unfavorable, there are still a few patients who demonstrate an extraordinary response to treatment with a prolonged progression-free survival (PFS); some patients even survive more than five years (5–7). But unfortunately, the intrinsic characteristics of these long-term survivors (LTS) are still unclear (5).

Previous studies have established that isocitrate dehydrogenase (IDH) mutation was a strong predictor associated with long-term survival of patients with GBM (8, 9), which has led the neuropathologists to reclassify GBM into two molecular subtypes: IDH-wildtype (IDH-wt) GBM and IDH-mutant (IDH-mut) GBM (10). Traditionally, IDH-mut GBM is regarded as a secondary malignancy that had transformed from a low-grade diffuse glioma (11). Meanwhile, IDH-wt GBM, which represents the major component (>90%) of the whole GBM cohort, is clinically defined as primary GBM (10). There are some studies that have explored the predictors for becoming a long-term survivor within the GBM population (5, 6, 8, 9). But up to now, no studies have compared the characteristics and clinical implications associated with LTS between IDH-wt and IDH-mut GBM.

Therefore, in the present study, we systematically compared the clinical, radiological and molecular characteristics between LTS and short-term survivors (STS) in the cohort of GBM. Furthermore, the intrinsic characteristics and clinical implications correlated with LTS between IDH-wt and IDH-mut GBM have also been explored. We found that the clinical, radiological, and molecular features of IDH-wt and IDH-mut LTS were significantly different and the two subtypes of LTS showed distinct PFS and post-progression survival (PPS), which can help the physicians to better understand GBM and may contribute to making more tailored treatment decisions for patients.

## Materials and Methods

### Patient Cohort

Forty-four patients who survived beyond five years after diagnosis of GBM (LTS) and 167 patients who survived less than one years after diagnosis of GBM (STS) between January 2007 and January 2015 in Beijing Tiantan Hospital affiliated to Capital Medical University were selected for inclusion in this study. Pathology review was performed by two experienced neuropathologists according to the 2016 World Health Organization (WHO) classification schema (10). All the patients, in our institution, once diagnosed with GBM are recommended to proceed with post-operative combination radiation and chemotherapy. However, there are still a small number of patients who missed out on chemotherapy or radiation for personal reasons. Radiation was performed within one month after operation, with a total dose of 60 Gy which was further divided into 30 fractions. The adjuvant chemotherapy regimen was mainly nimostine (ACNU) and temozolomide (TMZ), according to the previously described protocol (12). When tumor progressed, re-operation and reirradiation were performed if possible. Rechallenge with chemotherapy which commonly consisted of a combination of bevacizumab and temozolomide (TMZ) could be also attempted if patients showed relatively normal laboratory tests and maintained a reasonable performance status (ECOG: 0-2) (13).

### Radiological Evaluation

**Figure 1:**
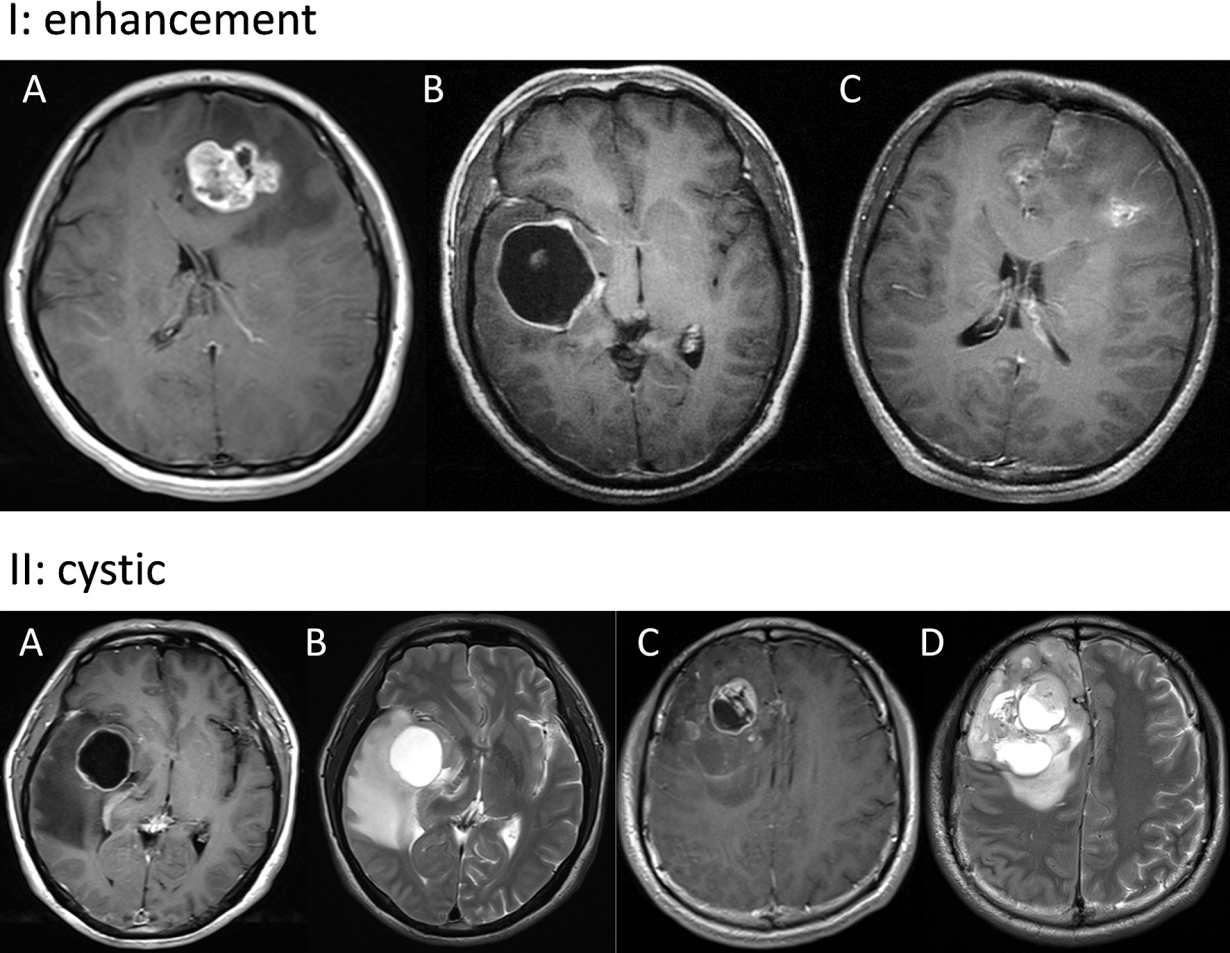
Panel I: Representative images of solid **(A)**, ring **(B)**, and irregular **(C)** enhancement. Panel II: Typical images of cystic GBM. A tumor in the right insular lobe with fluid-filled cysts **(A, B)** and a tumor in the right frontal lobe with large central necrosis **(C, D)**.

The radiological evaluation was performed by 3 experienced neuroradiologists who were blind to the clinical outcome of patients. Radiological features included tumor location, tumor size, enhancement, cystic change, and extent of resection. The calculation of tumor size was mainly based on the T1-weighted imaging (T1WI) contrast enhanced area. Enhancement was classified into three subtypes: solid, ring, irregular (**Figure 1**) (14). Solid subtype was relatively uniformly enhanced and concurrent with a well-circumscribed edge. Ring subtype was characterized by a ring-like enhancement with central necrosis or cyst. Irregular subtype had scattered enhancement which was irregularly shaped. A cystic tumor was defined as those with a large cyst cavity comprising at least half of the whole tumor and the cyst was filled with fluid that showed a radiographic appearance similar to cerebrospinal fluid on T2-weighted imaging (T2WI) (**Figure 1**) (15). Hence, tumors with fluid-filled cysts and those with large central necrosis were both regarded as cystic tumors in this study. Within 72 hours after operation, an enhanced magnetic resonance imaging (MRI) was carried out to assess the extent of resection (EOR). EOR was calculated on the basis of contrast-enhanced T1WI, according to the following equation: (preoperative tumor volume - postoperative tumor volume)/preoperative tumor volume.

### Molecular Biomarkers Detection

**Figure 2:**
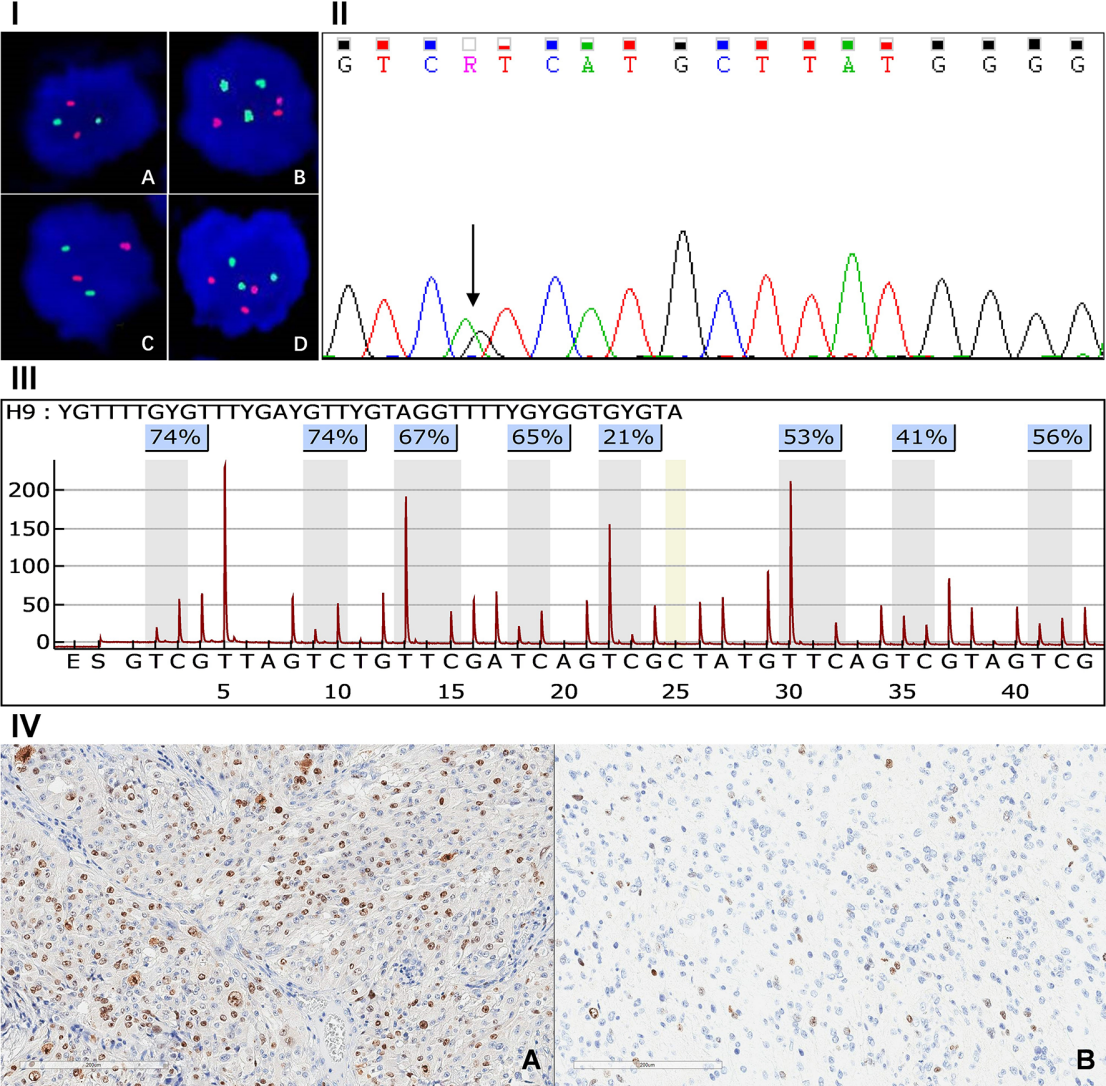
Panel I, FISH detection result of 1q/19p polysomy: 1p intact **(A)**, 1q polysomy **(B)**, 19q intact **(C)**, 19p polysomy **(D)**. Panel II: Sanger sequence of IDH1 mutation. Panel III: Assay of MGMT promoter methylation in GBM. Panel IV: Immunohistochemical staining of high **(A)** and low **(B)** Ki-67 index.

Abnormality of chromosome 1p/19q, IDH mutation and O^6^-methylguanine-DNA methyltransferase (MGMT) promoter methylation were respectively analyzed by fluorescence *in situ* hybridization (FISH) (16), Sanger sequencing (17) and pyrosequencing (18), according to previously described methods (**Figure 2**). Ki-67 index was detected by immunohistochemistry (IHC) staining which was done with a monoclonal mouse antibody (1:80 dilution, Dako) (**Figure 2**). The expression level of the Ki-67 index was defined as either high (≥30%) or low (<30%) for interpretation, according to the percentage of IHC-positive cells (19).

### Follow-Up

After operation, patients were regularly followed up with brain MRI scans until death. MRIs were performed at an interval of three months, or more frequent in the event of clinical changes, such as seizures or neurologic deterioration. Progression pattern was divided into local and non-local subtypes based on the preoperative and serial postoperative radiographic images (**Figure S1**). Local recurrence was those with lesion located in the resection cavity or in continuity with it, or less than 2 cm from the primary tumor margins, while the recurrence lesion border beyond 2 cm from the original cavity was defined as non-local failure (20). PFS was defined as the time period from the date of operation to the date of progression or recurrence demonstrated by MRI, death or last follow up. Overall survival (OS) was defined as the time period from the date of initial operation to the date of death or last follow-up. Timespan between the first progression and death/last follow-up was defined as post-progression survival (PPS). The median follow-up of this cohort was 71.5 (range: 1.0-130.0) months. There were 195 (92.4%) patients with progression and 176 (83.4%) patients had died by the time of data analysis.

### Statistical Analysis

All the analyses were performed with SPSS (version 22.0, Chicago, IL, USA) and R software (http://www.r-project.org, The R Foundation). Comparisons of categorical variables between the groups were performed using chi-square test or Fisher’s exact test, while differences in age at diagnosis, tumor size and Karnofsky performance scale (KPS) score were evaluated by student t-test. The variables with P values less than 0.05 were entered into the multivariate logistic regression analysis to identify the independent predictors of LTS. Survival rates were calculated using the Kaplan-Meier methods, and differences were compared by log-rank tests. All tests were two-sided, and difference with a *P* value less than 0.05 was considered to be statistically significant.

## Results

### Patient Population

We identified 211 patients, including 44 (20.9%) LTS and 167 (79.1%) STS. Of these 44 LTS, there were 17 (38.6%) patients with wildtype IDH and 27 (61.4%) patients with mutant IDH. Our cohort consisted of 126 males and 85 females with a mean age of 49.0 ± 11.8 years. All the clinical, radiological, and molecular characteristics of patients were summarized in **Table 1**.

**Table 1:** Comparisons of baseline characteristics between short- and long-term survivors.

Variables	All (n=211)	STS (n=167)	LTS (n=44)	*P* value
Age at diagnosis (years)	49.0 ± 11.8	49.9 ± 11.3	41.2 ± 11.1	<0.001
Gender				0.432
Male	126/211	102/167	24/44	
KPS score	80.0 ± 14.2	76.2 ± 14.9	82.2 ± 8.3	0.002
Tumor size (mm)	49.2 ± 18.9	49.5 ± 19.1	48.1 ± 18.3	0.652
Tumor location				0.039*
Frontal	86/211	60/167	26/44	
Temporal	56/211	45/167	11/44	
Parietal	35/211	32/167	3/44	
Occipital	18/211	16/167	2/44	
Others	16/211	14/167	2/44	
Laterality				0.138
Right	92/211	67/167	25/44	
Left	95/211	80/167	15/44	
Bilateral	24/211	20/167	4/44	
Enhancement				0.139
Solid	144/211	118/167	26/44	
Ring	38/211	30/167	8/44	
Irregular	29/211	19/167	10/44	
Cystic change				<0.001
Yes	57/211	32/167	25/44	
Extent of resection				<0.001
GTR	91/211	53/167	38/44	
Chemotherapy				0.084
Temozolomide	170/211	134/167	36/44	
Nimostine	27/211	19/167	8/44	
None	14/211	14/167	0/44	
Radiotherapy				0.005*
Yes	188/211	144/167	44/44	
Recurrence pattern				0.838
Local	156/195	134/167	22/28	
MGMT promotor				<0.001
Methylation	68/160	44/126	24/34	
IDH				<0.001
Mutation	55/211	28/167	27/44	
1q/19p co-polysomy				0.047
Yes	30/202	21/167	9/35	
Ki-67 index				0.510
High	70/193	59/158	11/35	

KPS, Karnofsky performance scale; GTR, gross-total resection; MGMT, O^6^-methylguanine-DNA-methyltransferase; IDH, isocitrate dehydrogenase.

*by Fisher exact test.

### Comparison of Baseline Characteristics Between STS and LTS

LTS patients were younger (41.2 ± 11.1 *vs.* 49.9 ± 11.3, *P *< 0.001), and had higher KPS score (82.2 ± 8.3 *vs.* 76.2 ± 14.9, *P *= 0.002) than the STS. But they shared a similar gender ratio and tumor size (*P *> 0.05). There were more frontal tumors in LTS (59.1% *vs.* 35.9%, *P *= 0.039), but no significant difference has been observed in the laterality (*P *= 0.138). The enhancement features were also similar between STS and LTS (*P *= 0.139), while LTS showed a higher frequency of cystic change (56.8% *vs.* 19.2%, *P *< 0.001). With respect to the treatment information, LTS patients were more likely to have undergone gross-total resection (GTR) (86.4% *vs.* 31.7%, *P *< 0.001), and to have received chemotherapy (100.0% *vs.* 91.6%, *P *= 0.084) and radiotherapy (100.0% *vs.* 86.2%, *P *= 0.005), compared with the STS. The molecular profile of LTS patients was characterized by a higher rate of MGMT promoter methylation (70.6% *vs.* 34.9%, *P*<0.001), IDH mutation (61.4% *vs.* 13.2%, *P *< 0.001), and 1q/19p co-polysomy (25.7% *vs.* 12.6%, *P *= 0.047). The Ki-67 index was similar among groups (*P *= 0.510) (**Table 1**).

On the basis of these predictors identified by univariate analyses, a multivariate logistic regression model was built. The final results showed that age < 50 years (odds ratio [OR] = 1.081, 95% confidence interval [CI]: 1.022-1.141, *P *= 0.006), KPS score ≥ 70 (OR = 22.354, 95% CI: 2.028-246.449, *P *= 0.011), cystic change (OR = 3.791, 95% CI: 1.082-13.275, *P *= 0.037), GTR (OR = 18.731, 95% CI: 4.636-75.690, *P *< 0.001), MGMT promoter methylation (OR = 5.553, 95% CI: 1.591-19.379, *P *= 0.007), and IDH mutation (OR = 4.321, 95% CI: 1.007-18.535, *P *= 0.049) were confirmed as predictive factors for LTS (**Table 2**).

**Table 2:** Results of multivariate logistic regression analysis.

Variables	Odds ratio (OR)	95% Confidence interval (CI)	*P* value
Age at diagnosis			
<50 years	1.081	1.022-1.141	0.006
KPS score			
≥70	22.354	2.028-246.449	0.011
Cystic change			
Yes	3.791	1.082-13.275	0.037
Extent of resection			
GTR	18.731	4.636-75.690	<0.001
MGMT promotor			
Methylation	5.553	1.591-19.379	0.007
IDH			
Mutation	4.321	1.007-18.535	0.049

KPS, Karnofsky performance scale; GTR, gross-total resection; MGMT, O^6^-methylguanine-DNA-methyltransferase; IDH, isocitrate dehydrogenase.

### Comparison of Baseline Characteristics Between IDH-Wt and IDH-Mut LTS

Compared with the IDH-wt LTS, IDH-mut LTS had a lower rate of solid enhancement (44.4% *vs.* 82.3%, *P *= 0.036), but higher rates of cystic change (70.4% *vs.* 35.3%, *P *= 0.022), local recurrence (95.0% *vs.* 37.5%, *P *= 0.003), and 1q/19p co-polysomy (44.4% *vs.* 5.9%, *P *= 0.018). The Ki-67 index of IDH-mut LTS was lower than that in the IDH-wt LTS, but the difference was not statistically significant (16.7% *vs.* 47.1%, *P *= 0.053) (**Table 3**).

**Table 3:** Comparisons of baseline characteristics between IDH-wt and IDH-mut long-term survivors.

Variables	IDH-wt (n=17)	IDH-mut (n=27)	*P* value
Age at diagnosis (years)	41.1 ± 12.3	41.3 ± 10.5	0.938
Gender			0.651
Male	10/17	14/27	
KPS score	80.0 ± 8.7	84.7 ± 7.4	0.115
Tumor size (mm)	52.4 ± 16.3	45.3 ± 19.2	0.213
Tumor location			0.351
Frontal	10/17	16/27	
Temporal	6/17	5/27	
Parietal	0/17	3/17	
Occipital	1/17	1/27	
Others	0/17	2/27	
Laterality			0.342
Right	8/17	17/27	
Left	8/17	7/27	
Bilateral	1/17	3/27	
Enhancement			0.036
Solid	14/17	12/27	
Ring	2/17	6/27	
Irregular	1/17	9/27	
Cystic change			0.022
Yes	6/17	19/27	
Extent of resection			0.380*
GTR	16/17	22/27	
Chemotherapy			0.125*
Temozolomide	16/17	20/27	
Nimostine	1/17	7/27	
Radiotherapy			NA
Yes	17/17	27/27	
Recurrence pattern			0.003*
Local	3/8	19/20	
MGMT promotor			1.0
Methylation	12/17	12/17	
1q/19p co-polysomy			0.018*
Yes	1/17	8/18	
Ki-67 index			0.053
High	8/17	3/18	

IDH, isocitrate dehydrogenase; KPS, Karnofsky performance scale; GTR, gross-total resection; MGMT, O^6^-methylguanine-DNA-methyltransferase; NA, not applicable.

*by Fisher exact test.

### Differential Clinical Implications Between IDH-wt and IDH-Mut LTS

**Figure 3:**
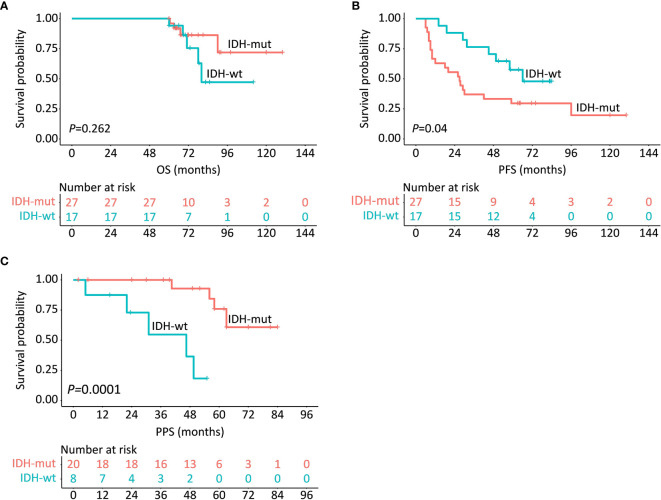
Survival comparisons in regards to OS **(A)**, PFS **(B)** and PPS **(C)** between IDH-wt and IDH-mut LTS.

With respect to the prognostic implication of IDH in LTS patients, the survival rates between IDH-wt and IDH-mut subgroups in terms of PFS, OS, and PPS were compared. Results demonstrated that IDH-wt LTS shared a similar survival rate with IDH-mut LTS in regard to OS (*P *= 0.262) (**Figure 3A**). But interestingly, the median PFS of IDH-wt LTS was unexpectedly longer than that of IDH-mut LTS (66.0 *vs.* 27.0 months, *P *= 0.040). Conversely, the median PPS of IDH-wt LTS was significantly shorter than that of IDH-mut LTS (46.5 months *vs.* not reached, *P *= 0.0001) (**Figures 3B, C**).

**Figure 4:**
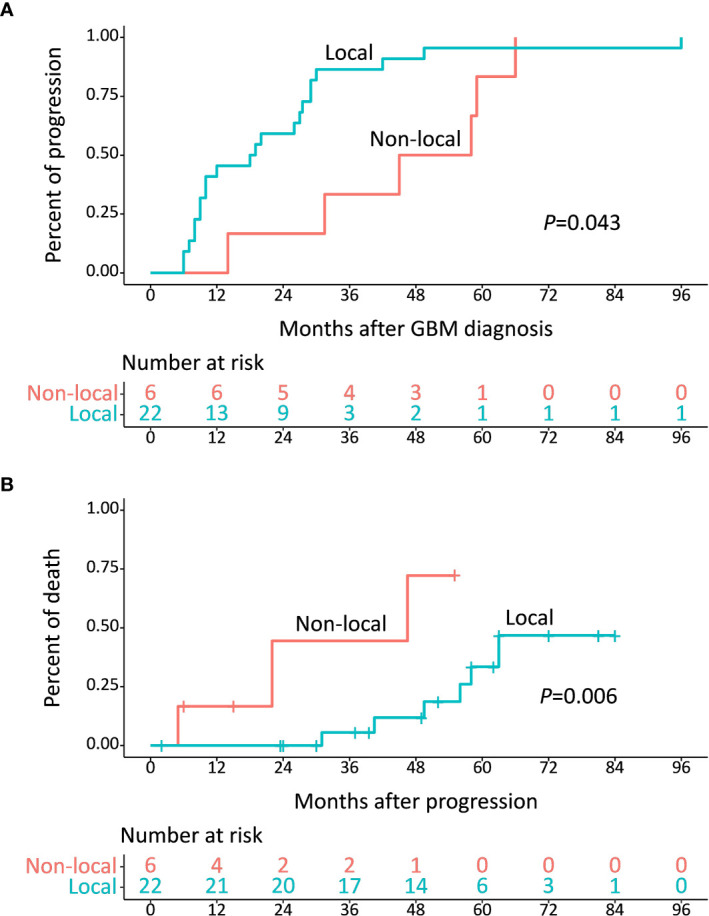
**(A)** Median time from diagnosis to development of progression was 18.5 months for local recurrence and 45.0 months for non-local recurrence (*P* = 0.043). **(B)** Median survival after diagnosis of non-local recurrence was 46.5 months, which was shorter than that of local recurrence (*P* = 0.006).

Considering the distinct survival distribution in PFS and PPS between IDH-wt and IDH-mut LTS, we explored the clinical implication of recurrence pattern in LTS patients. Of these 44 LTS, 22 patients (including 3 IDH-wt LTS and 19 IDH-mut LTS) experienced local recurrence at a median period of 18.5 (10.0-29.0) months and 6 patients (including 5 IDH-wt LTS and 1 IDH-mut LTS) experienced non-local recurrence at a median period of 45.0 (16.2-76.8) months, which imparted a significant difference (*P *= 0.043) (**Figure 4A**). Moreover, the percent of death was also markedly different between patients with local and non-local recurrence (*P *= 0.006) (**Figure 4B**).

**Figure 5:**
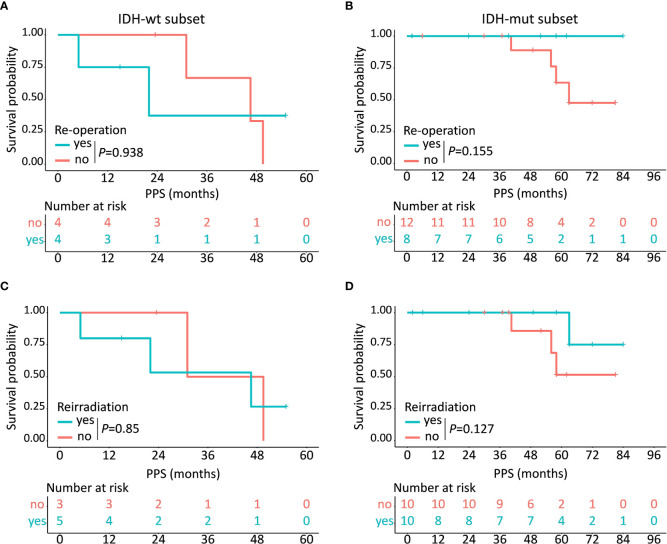
Prognostic implications of different kinds of treatment regimens in IDH-wt and IDH-mut subtypes. In the subset of IDH-wt LTS, no obvious clinical benefit has been observed after receiving re-operation **(A)** or reirradiation **(C)** while IDH-mut LTS show a trend towards increased survival after receiving re-operation **(B)** and reirradiation **(D)**.

Of the 28 patients who underwent tumor progression, 12 (42.9%) patients (including 4 IDH-wt LTS and 8 IDH-mut LTS) received re-operation, 15 (53.6%) patients (including 5 IDH-wt LTS and 10 IDH-mut LTS) received reirradiation, and all (100.0%) patients (including 8 IDH-wt LTS and 20 IDH-mut LTS) received rechallenge with chemotherapy (**Figure S2**). Kaplan-Meier plots demonstrated that IDH-mut LTS showed a trend towards increased survival after receiving re-operation (*P *= 0.155) and reirradiation (*P *= 0.127), while this clinical benefit disappeared in the subset of IDH-wt (*P*>0.05) (**Figure 5**).

## Discussion

GBM is the most aggressive intracranial malignancy, with rapid growth, inevitable recurrence and high mortality (4). Only a small fraction of patients can achieve a long-term survival after surgical resection and chemoradiotherapy. But the diagnostic threshold of LTS varies significantly in the pre-existing literatures, ranging from 2 years to 5 years (5, 9, 18, 21). As the median survival of patients with GBM is about 1 year and 5-years survival rate is regarded as a predictor of better tumor control, patients who survived beyond 5 years after diagnosis, in this study, were identified as LTS, while those with a survival less than 1 year were defined as STS (5). We systematically compared the clinical, radiological, and molecular features between STS and LTS. Although the characteristics of LTS in GBM have been widely investigated (5, 6, 8, 9, 18, 21, 22), there is no study devoted to exploring the intrinsic properties of IDH-wt and IDH-mut LTS. To our knowledge, it’s the first study that was aimed to disclose the differential predictors and clinical implications between IDH-wt and IDH-mut LTS. Our results demonstrated that IDH-mut LTS had a lower rate of solid enhancement, but higher rates of cystic change, 1q/19p co-polysomy, and local recurrence. IDH-mut LTS showed a shorter PFS, but a significantly prolonged PPS than those of IDH-wt LTS.

Younger age, better performance status, and higher resection degree are universally regarded as predictors of superior survival in GBM (5, 23–27). In this study, we found that LTS were a subgroup of patients who had a younger age, a higher KPS score, and a more radical resection. In 2014, Field et al. compared the characteristics between patients who survived more than 2 years and those with a survival less than 6 months (23). Final results showed that younger age, better performance status, gross macroscopic resection, and clinical trial participation were independent predictors of LTS (23). Similarly, some authors have explored the features of LTS who were defined as surviving over 3 years by comparing with controls and found that LTS were younger, had better performance status, and were more likely to have received GTR and adjuvant chemotherapy (24, 25). These studies, however, enrolled a small number of LTS, which to a certain extent decreased the reliability of conclusions. Recently, a report of 2249 LTS from National Cancer Database maintained that factors associated with improved 5-year survival were younger age, female gender, less medical comorbidities, non-white race, higher salary, left-sided tumors and tumors outside the brainstem, and radiotherapy (5). However, the molecular parameters have not been explored in the study.

In addition to the clinical factors, the molecular biomarkers, such as IDH and MGMT, are also closely correlated with patient’s survival (18, 24, 28–30). Most of the prior studies repeatedly demonstrated that IDH mutation was a prognostic factor associated with prolonged survival (18, 31). GBM was divided into two major categories based on the status of IDH since 2016 when the latest World Health Organization classification schema of brain tumor was issued (10). In our study, the frequencies of IDH mutation and MGMT promoter methylation in LTS were significantly higher than those in STS, which was consistent with previous findings (18). Barbus et al. found that IDH mutation was more prevalent in LTS (28). However, in a larger cohort study of comparative patients, the presence of IDH mutation was not significantly associated with LTS (9). Of note, their further analysis showed that significantly more LTS were MGMT methylated and IDH mutant (9). MGMT is a DNA repair protein involved in reversing methylation damage from alkylating agent (32). It’s universally acknowledged that methylated MGMT is linked to increased chemosensitivity and generally confers an improved survival (32). The frequency of MGMT promoter methylation of LTS was 70.6% in our study, which was in accordance with prior results (6, 9, 24, 29, 30). It is well-established that methylated MGMT is more prevalent in LTS of GBM, compared with the STS patients (18, 24, 29, 30). Together these suggest that MGMT methylation is one of the most important features of LTS.

Within the group of LTS, solid enhancement seemed to be more likely occurred in IDH-wt LTS, while cystic change was predominant in IDH-mut group. Rathore and colleagues divided GBM into three distinct subtypes based on the signature of enhancement and found that classical tumors were more prevalent in the solid subtype which showed the worst clinical outcome (14). Cystic change is confirmed as a prognostic factor associated with favorable outcomes (33, 34). Some authors held that cystic GBM may develop from malignant transformation of a previously undiagnosed cystic low-grade glioma (34). This explained why cystic change occurred more frequently in IDH-mut GBM cases which had a history of low-grade glioma within our cohort. Utsuki et al. (34) believed cystic GBM was less aggressive and had little infiltration of the peritumoral brain tissue, which is consistent with the lower Ki-67 index demonstrated in the tumors of the IDH-mut GBM group in our study. Furthermore, we found IDH-mut LTS presented with a higher rate of 1q/19p co-polysomy than IDH-wt GBM. In 2017, Zeng et al. analyzed the prognostic implication of 1q/19p polysomy in two large cohorts of astrocytic gliomas and found co-polysomy was an independent prognostic factor associated with prolonged survival (35). All the findings imply that IDH-mut GBM seems to be a less aggressive brain tumor, compared with IDH-wt GBM.

The most interesting finding of our study was that IDH-wt LTS showed a significantly higher rate of non-local failure compared with that in IDH-mut group, which determined the different survival distribution spectrum between IDH-wt and IDH-mut LTS. As we all know, non-local failure is a fatal event which commonly occurs later than local failure (20). In our study, the median time period from diagnosis to local failure was 18.5 months, which was shorter than the interval between diagnosis and non-local failure. Meanwhile, IDH-wt LTS had a higher rate of non-local failure than that of IDH-mut LTS. Therefore, the favorable PFS among IDH-wt LTS could be ascribed to a higher rate of non-local failure. Notably, although IDH-wt LTS conferred a longer PFS, the PPS of these patients was significantly shorter than IDH-mut LTS. With an attempt to interpret the opposite result observed in PPS, we explored the relationship between post-progression treatments and PPS. Finally, the survival analyses demonstrated that IDH-mut LTS showed a trend towards increased survival after receiving re-operation and reirradiation, while the clinical benefits disappeared in the subset of IDH-wt. Hence, non-local failure can be regarded as an endpoint event that predicts a poor treatment response.

There are several limitations of this study. Firstly, the fact that it is a retrospective study, means that there has been bias relating to our patient selection. Secondly, given the wide confidence intervals in some subgroups, our results should be interpreted with caution. Additionally, we should continue this study until the last patient reached the endpoint in order to recheck and confirm the results and conclusions in the future. Finally, functional and employment status of LTS in addition to cognition was not recorded which was of great importance in terms of assessing the quality of life (36).

## Conclusions

Despite improvements of median survival achieved in recent years, the percentage of patients surviving more than five years after diagnosis of GBM remains low. IDH-wt and IDH-mut LTS are two distinct subgroups which differ radically in terms of clinical, radiological, and molecular characteristics. Our findings provide a new approach for physicians to better understand the IDH-wt and IDH-mut GBM, which may contribute to developing more tailored therapeutic strategies for patients.

## Data Availability Statement

The original contributions presented in the study are included in the article/**Supplementary Material**. Further inquiries can be directed to the corresponding author.

## Ethics Statement

The studies involving human participants were reviewed and approved by Institutional Review Board of Capital Medical University. The patients/participants provided their written informed consent to participate in this study.

## Author Contributions

Acquisition of data: HJ, ML, CY, XZ, and QZ. Analysis and interpretation of data: HJ, KY, YC, and XR. Statistical analysis: HJ and KY. Drafting the article: HJ and SL. Funding acquisition: SL and YC. Conception and design: HJ, GZ, and SL. Study supervision: SL. All authors contributed to the article and approved the submitted version.

## Funding

This work was supported by the National Natural Science Foundation of China (81771309) and the Capital’s Funds for Health Improvement and Research (2020-2-1075).

## Conflict of Interest

The authors declare that the research was conducted in the absence of any commercial or financial relationships that could be construed as a potential conflict of interest.

## Supplementary Material

Supplementary Figure 1 Panel I: Representative images of local recurrence **(A–C)**. Preoperative image showed a lesion in the left frontal lobe, which was totally removed during operation. While 38 months after operation, the tumor recurred at the resection cavity. Panel II: Representative images of non-local recurrence **(A–F)**. Preoperative images showed a lesion in the left temporal lobe, which was totally removed during operation. After 45 months follow-up, a new non-enhanced lesion far from the original resection cavity was found in the corpus callosum.

Supplementary Figure 2 No significant difference was observed between IDH-wt and IDH-mut LTS in the number of patients who received re-operation **(A)**, reirradiation **(B)**, and rechallenge with chemotherapy **(C)**. NA, not applicable.
