# Supplementary figures and images for: Differential Predictors and Clinical Implications Associated With Long-Term Survivors in IDH Wildtype and Mutant Glioblastoma
Source: Front Oncol. 2021 May 13;11:632663. doi: 10.3389/fonc.2021.632663 (PMC8155513; doi:10.3389/fonc.2021.632663)

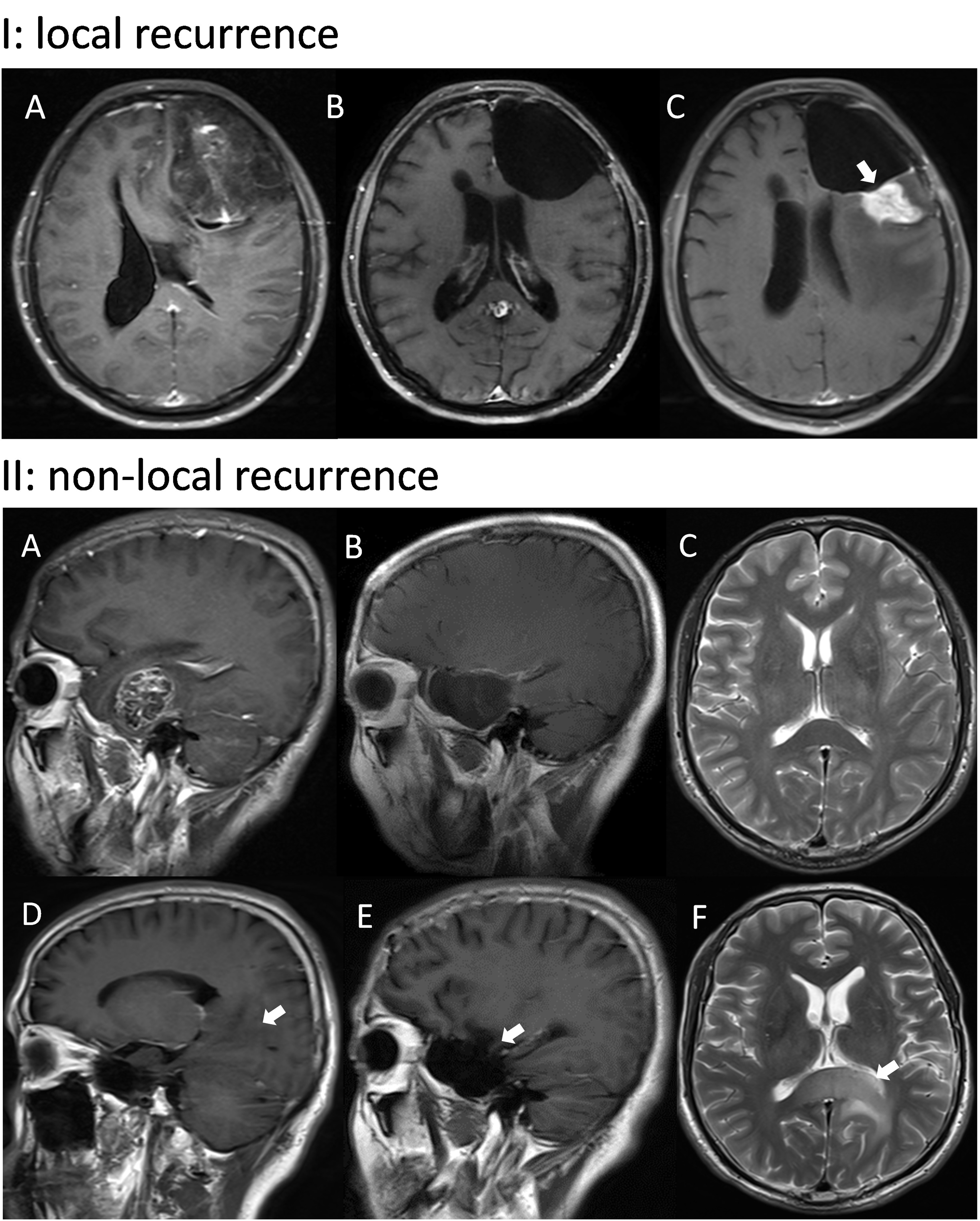

Supplement: Supplementary Figure 1 — Panel I: Representative images of local recurrence (A–C). Preoperative image showed a lesion in the left frontal lobe, which was totally removed during operation. While 38 months after operation, the tumor recurred at the resection cavity. Panel II: Representative images of non-local recurrence (A–F). Preoperative images showed a lesion in the left temporal lobe, which was totally removed during operation. After 45 months follow-up, a new non-enhanced lesion far from the original resection cavity was found in the corpus callosum. [file Image_1.jpeg]

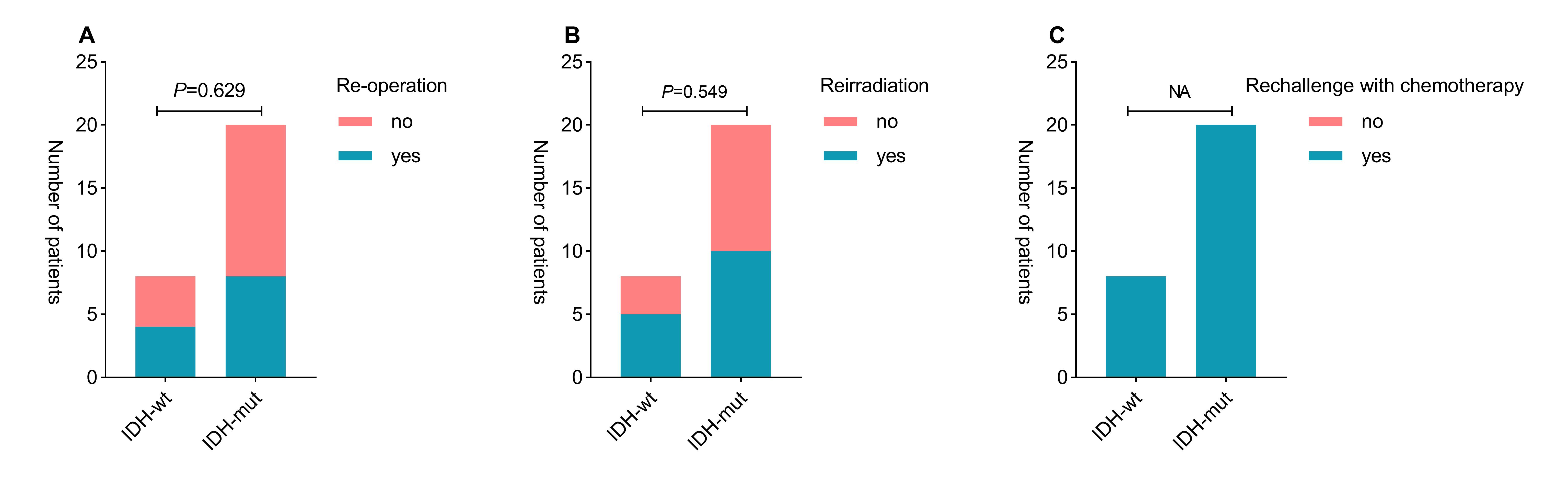

Supplement: Supplementary Figure 2 — No significant difference was observed between IDH-wt and IDH-mut LTS in the number of patients who received re-operation (A), reirradiation (B), and rechallenge with chemotherapy (C). NA, not applicable. [file Image_2.jpeg]
